# National Cancer Control Programmes in Europe in 2024: State of Play and Alignment with the European Guide for Quality NCCPs

**DOI:** 10.2478/sjph-2026-0014

**Published:** 2026-06-01

**Authors:** Marjeta Kuhar, Tit Albreht, Polona Kastelic, Ana Molina-Barceló, Oscar García Sauret, Marjetka Jelenc

**Affiliations:** National Institute of Public Health, Trubarjeva ulica 2, 1000 Ljubljana, Slovenia; Faculty of Social Sciences, University of Ljubljana, Kardeljeva ploščad 5, 1000 Ljubljana, Slovenia; Faculty of Medicine, University of Ljubljana, Vrazov trg 2, 1000 Ljubljana, Slovenia; FISABIO - Fundación para el Fomento de la Investigación Sanitaria y Biomédica de la Comunidad Valenciana, Avenida Cataluña 21, 46020 Valencia, Spain; Faculty of Health Sciences, University of Maribor, Žitna ulica 15, 2000 Maribor, Slovenia

**Keywords:** Cancer control, OriON Joint Action, National Cancer Control Programmes, Oncology, Cancer, Europe, obvladovanje raka, OriON Joint Action, Nacionalni programi za obvladovanje raka, onkologija, rak, Evropa

## Abstract

**Introduction:**

National Cancer Control Programmes (NCCPs) are essential policy documents guiding national cancer control planning. Their existence and quality are crucial for reducing cancer incidence, morbidity, and mortality, and for improving the quality of life of people with cancer and the population. The main aim of the present research, conducted within OriON Joint Action (2024–2025), was to explore and analyse the state of play, type, duration, evaluation, and quality of NCCPs across the European Union (EU) and selected countries, with a focus on the inclusion of key elements outlined in the European Guide for Quality NCCPs (Guide).

**Methods:**

A structured survey was prepared, validated, and carried out in 2024 across 34 countries, including EU Member States, Iceland, Montenegro, Norway, Turkey, and Ukraine. The results were analysed and presented in tables and figures. Descriptive analysis using frequencies and percentages was conducted.

**Results:**

The response rate was 100%. Of the 34 participating countries, 31 reported having an NCCP or equivalent cancer control document in 2024. Most countries had 1 comprehensive document, while 9 prepared multiple documents. The majority were defined as programmes and/or strategies, followed by plans and policies. In terms of quality, as recommended by the Guide, only 6 countries fully incorporated all suggested elements into their NCCPs.

**Conclusions:**

Our findings indicate that most European countries recognise the significant challenge posed by cancer as a major public health problem and have developed NCCPs or equivalent cancer control documents. However, in terms of quality, there remains considerable room for improvement.

## Izvleček

**Uvod:**

Nacionalni programi za obvladovanje raka (NPOR) so ključni strateški dokumenti, ki usmerjajo nacionalno načrtovanje in izvajanje politik obvladovanja raka. Njihova priprava in kakovost sta bistvena za zmanjševanje incidence, obolevnosti in umrljivosti zaradi raka ter za izboljševanje kakovosti življenja bolnikov z rakom in prebivalstva. Namen pričujoče raziskave, ki je bila izvedena v sklopu projekta OriON Joint Action (2024–2025) je bil proučiti in analizirati stanje glede obstoja NPOR v državah Evropske unije (EU) in izbranih državah, njihovo število, tip, trajanje, evalvacijo, financiranje in kakovost, s posebnim poudarkom na vključenosti elementov, ki zagotavljajo kakovost, opredeljenih v Evropskem vodniku za kakovostne NPOR (Vodnik).

**Metode:**

Leta 2024 je bil pripravljen in validiran vprašalnik ter poslan ekspertom s področja NPOR v 34 državah, vključno z državami članicami EU, Islandijo, Črno goro, Norveško, Turčijo in Ukrajino. Rezultati so bili analizirani in predstavljeni v tabelah in slikah. Izvedena je bila deskriptivna analiza po frekvencah in odstotkih.

**Rezultati:**

Eksperti s področja NPOR so se izjemno dobro odzvali na vprašalnik, stopnja odziva je bila kar 100-odstotna. Od 34 sodelujočih držav je 31 držav poročalo, da imajo pripravljen NPOR ali enakovreden dokument za obvladovanje raka. Večina držav je imela v letu 2024 po en NPOR, medtem ko jih je 9 pripravilo več dokumentov, ki urejajo področje obvladovanja raka. Najpogosteje so bili ti dokumenti oblikovani kot programi in/ali strategije, sledili so načrti in politike. Glede veljavnosti dokumentov se je kar 14 držav (45 %) odločilo za daljše obdobje – od 7 do13 let. Več kot polovica držav (20 držav; 64 %) je odločenih, da bodo NPOR obnovile, oz. ponovno pripravile pred potekom trenutnega veljavnega dokumenta. Več kot dve tretjini držav (24 držav; 78 %) svoj NPOR spremlja in evalvira ob vključitvi različnih institucij in indikatorjev. V vsebinskem smislu – skladno s priporočili Vodnika, ki obsega deset tematskih poglavij, razdeljenih v tri sklope – je le šest držav v svoje NPOR v celoti vključilo vse predlagane elemente. Nezadostno vključena področja so predvsem financiranje, spremljanje bremena raka, viri in upravljanje.

**Zaključki:**

Ugotovitve analize, pripravljene na podlagi vprašalnika v sklopu projekta OriON Joint Action v letu 2024 so pokazale, da večina evropskih držav prepoznava raka kot pomemben družbeni in zdravstveni izziv ter velik javnozdravstveni problem in je pripravila NPOR ali enakovredne dokumente za obvladovanje raka. Vendar pa na področju kakovosti obstaja veliko prostora za izboljšave, saj je le šest držav v svoje NCCP vključilo vsa tematska področja, ki zagotavljajo kakovost in so priporočena v Vodniku.

## 1 INTRODUCTION

National Cancer Control Programmes (NCCPs) are key documents in cancer control management and planning. They are defined by the World Health Organization (WHO) as ‘public health programmes designed to reduce cancer incidence and mortality and improve the quality of life of people with cancer, through the systematic and equitable implementation of evidence-based strategies for the prevention, early detection, diagnosis, treatment, and palliation, making the best use of available resources (1). As the management of cancer control is complex and effective planning is essential, NCCPs represent useful tools for supporting health systems in responding to the challenges of cancer (2, 3).

Over the past 30 years, there has been a gradual uptake of cancer control programmes in many countries worldwide (4, 5). However, the European Union (EU) has produced some of the most innovative and pioneering initiatives in the field of NCCPs. England and France were among the countries that paved the way for the EU-wide discussion on the need to establish national or regional cancer programmes, plans, or strategies in all EU Member States (MSs) (2, 6, 7). This led to the gradual development of NCCPs across the EU (8).

Recognising cancer as an important public health challenge, the European Parliament and Council urged the European Commission (EC) to take strong action in supporting MSs in the fight against cancer. In 2009, the EU recommended that its MSs develop NCCPs or strategies by 2013 (9,10,11). The EC supported policy development through projects called Joint Actions (JAs): EPAAC JA, 2011–2013, CanCon JA, 2014–2017, iPAAC JA, 2018–2021, and OriON JA, 2024–2025 (12,13,14,15,16). JA projects are collaborative, EU-funded initiatives in which national health authorities from multiple countries work together to address key health policy priorities. Implemented under the EU4Health Programme, these projects share knowledge, test tools, and develop common approaches that are more effective than individual national actions.

In all JAs, the area of NCCPs has been an focus area, with the preparation of NCCPs and their quality being explored (17,18,19,20). In response to the EC’s call to develop NCCPs, within EPAAC JA, the European Guide for Quality NCCPs (Guide) was prepared and published in 2015. It is a unique document of this type, developed with the intention of serving as an operational tool for policymakers in the preparation of NCCPs, as well as to support the inclusion of all relevant areas and topics that a quality NCCP should cover (21).

In 2021, the EC presented Europe’s Beating Cancer Plan (EBCP), the umbrella document for cancer control in the EU. EBCP aims to reduce the burden of cancer, which represents a major public health problem in most European countries. The document is structured around 4 key action areas: prevention, early detection, diagnosis and treatment, and the quality of life of people with cancer and survivors. Additionally, it comprises cross-cutting themes, including research and innovation, digital and personalised medicine, and 10 flagship initiatives. OriON JA is based on the Flagship Initiative No 9 and addresses cancer inequalities (22). Twenty-three partners from 17 EU MSs and selected countries participated in the project (Belgium, Croatia, Cyprus, Greece, Hungary, Ireland, Italy, Lithuania, Malta, the Netherlands, Norway, Poland, Romania, Slovakia, Slovenia, Spain, and Sweden). The goals of the project were to carry out an analysis of the state of play of NCCPs, with a special focus on cancer inequalities, and to support the monitoring of the implementation of EBCP in MSs’ cancer policy documents (16).

The aim of this paper is to present the main findings from the analysis of the 2024 survey on NCCPs conducted within OriON JA in Europe, , with an emphasis on the state of play and alignment with the Guide.

## 2 METHODS

To obtain key information on NCCPs, their state of play and quality, a retrospective observational study was conducted in 2024 using a structured survey. Project partners from participating countries within the OriON JA and the European Observatory on Health Systems and Policies were entrusted with reviewing the first draft of the survey on NCCPs, prepared by the lead partners of the task (National Institute of Public Health-NIPH, Slovenia, and Fisabio, Spain). The survey, which was validated by a methodologist and 3 international experts in the field of cancer control, was intended to provide the state of play regarding the NCCPs and their quality, and the coverage of the main areas and elements recommended by the Guide (21) in the EU, Iceland, Montenegro, Norway, Turkey, and Ukraine in 2024.

The survey questions were divided into 3 sections: state of play, quality of NCCPs, and cancer inequalities. For the present analysis, only topics addressing the state of play regarding NCCPs and their quality were examined. In the survey, there were 14 general questions and 1 question about the quality of NCCP content. The topics of the first 2 sections of the survey presented in this article are listed in Table 1. The survey questions are fully detailed in the dataset linked to this article (23), which also summarises the responses and is openly available.

**Table 1.** Sections and topics of the survey.

**Sections (survey questions)**	**Topics**
**Section 1 – State of play (Q1–Q14)**	Existence, type, and number of cancer documents, availability, integration of cancer control into other policies, duration of validity of the NCCP, intention to renew the NCCP, evaluation of the NCCPs, use of indicators, inclusion of indicators in the NCCPs, funding.
**Section 2 – Quality (Q15)**	Primary prevention; cancer screening; early detection, diagnosis, and treatment; cancer data and information; research; psychosocial oncology care; health promotion; palliative and end-of-life care; access to innovative cancer treatment; education and training; survivorship; patient empowerment; governance; cancer resources; monitoring of cancer burden; financing.

Legend: NCCP - National Cancer Control Programme

The terms programme, plan, strategy, and policy were explained in the survey, which is available in the dataset linked to this article (23). A programme is a set of activities addressing desired changes; a plan is a concrete arrangement for a specific purpose; a strategy outlines the mission and approach; and a policy describes overarching goals for cancer control.

The coordinator (NIPH) compiled the list of experts responsible for NCCPs in the participating countries. The survey, including individualised cover letters tailored to each participating country context, was sent at the end of April 2024 using the 1KA online platform to the identified national experts from Austria, Belgium, Bulgaria, Croatia, Cyprus, Czech Republic, Denmark, Estonia, Finland, France, Germany, Greece, Hungary, Iceland, Ireland, Italy, Latvia, Lithuania, Luxembourg, Malta, Moldova, Montenegro, the Netherlands, North Macedonia, Norway, Poland, Portugal, Romania, Slovak Republic, Slovenia, Spain, Sweden, Turkey, and Ukraine. The experts were kindly asked to complete the survey within 3 weeks.

Completed surveys were analysed, and results that represent the main input for future policy recommendations aimed at improving the situation regarding NCCPs were prepared. Descriptive analysis using frequencies and percentages was conducted for each survey question. The analysis was conducted independently by an experienced policy analyst, and the findings were validated through joint review and discussion with the work package leader and project partners. The detailed results are available in the dataset linked to this article (23), while the main findings are presented in the figures.

The key types of evaluation, according to the Guide (21), are evaluation by structure, process, and outcomes. Structure refers to the setup of a system, including resources, organisation, and infrastructure. Process relates to the activities taken to deliver services, including procedures and strategies. Outcomes represent the results of these processes, such as improvements in health, patient satisfaction, or system efficiency. These evaluation typologies served as the analytical framework for classifying the indicators used to monitor and evaluate the NCCPs presented in Figure 2.

## 3 RESULTS

**Figure 1.**
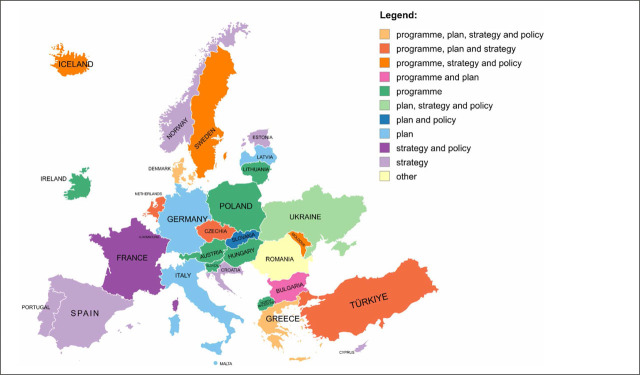
Types of cancer control documents in 31 participating countries.

The analysis is based on responses from 31 countries (91.2%) that confirmed the existence of an NCCP or an equivalent cancer control document. Most of these countries (22 out of 31; 71.0%) reported having a single document addressing cancer control. In contrast, 9 countries (29.0%) indicated that their cancer control is addressed through several documents. Regarding the type of documents, 16 countries reported having a programme, 16 a strategy, and 13 a plan. A policy was reported less frequently, with only 8 countries reporting its presence. In addition, more than half of the countries (17 out of 31; 54.8%) reported having supplementary policy documents addressing cancer control. Types of cancer control documents by country are presented in Figure 1.

The largest group of countries (11 out of 31; 35.5%) reported that their current NCCPs expire in 2030. Regarding the duration of the cancer documents, most countries favour medium- to long-term planning horizons. A duration of 3–5 years was reported by 10 countries (32.3%), while longer durations of 7–13 years were reported by 14 countries (45.2%). A smaller number of countries indicated that their cancer control documents have a flexible or open-ended duration.

Most countries (20 out of 31; 64.5%) reported an intention to update/renew their NCCPs upon expiry of the current document. Only 1 country explicitly stated that it has no intention to update/renew the current cancer control document. Several countries reported obstacles to the development or implementation of their NCCPs and/or relevant cancer control documents, citing systemic challenges such as limited institutional support or competing health policy priorities.

**Figure 2.**
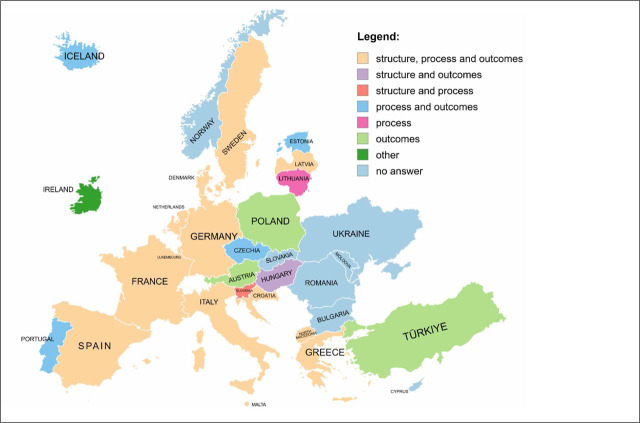
Evaluation typologies of NCCPs in 31 participating countries.

The survey results indicate that 7 out of the 31 participating countries (22.6%) reported that their NCCPs and/or relevant cancer control documents are not subject to systematic evaluation. In contrast, 24 countries (77.6%) reported having formal evaluation processes in place. These processes involve institutions within national health authority structures, primarily ministries of health, as well as specialised advisory or coordinating bodies supporting cancer control governance. A majority of participating countries (19 out of 31; 61.3%) reported using indicators to monitor implementation progress and/or evaluate outcomes of their NCCPs. The evaluation typologies reported by 24 countries that responded to the question on evaluation are presented in Figure 2.

Mid-term and final evaluations of NCCPs and/or respective cancer control documents were reported by 8 countries (25.8%), while final evaluations only were reported by 4 countries (12.9%). In addition, more than one third (12 countries) reported using alternative forms of evaluation. More than half of the participating countries included indicators in their NCCPs and/or relevant cancer control documents.

The survey also examined the availability of funding for implementing NCCPs. The results indicate that fewer than half of the participating countries (14 out of 31; 45.2%) reported having secured funding for implementing their NCCPs, whereas a majority (16 out of 31; 51.6%) reported ongoing challenges in securing such funding.

**Figure 3.**
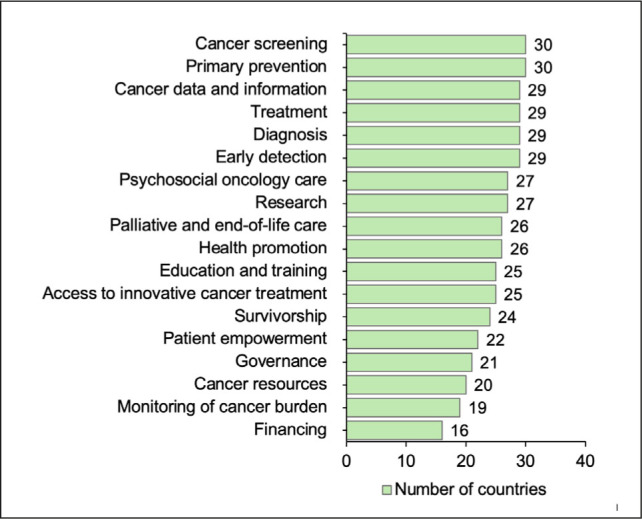
Inclusion of key elements of a quality NCCP across 31 participating countries.

With regard to the quality of NCCPs, the areas most frequently addressed in NCCPs and/or relevant national cancer control documents, as recommended by the Guide, were primary prevention, cancer screening, early detection, diagnosis, treatment, cancer data and information systems, psychosocial oncology care, and education and training. More than 80% of countries with an operational NCCP reported including these areas in their NCCPs and/or relevant cancer control documents. In contrast, several key areas were not included adequately. These areas include financing, cancer resources (including workforce and other resources), monitoring of cancer burden, governance, patient empowerment, and survivorship. The number of countries that reported including the listed key elements of a quality NCCP in their NCCPs and/or relevant cancer control documents is presented in Figure 3. Six countries included all key elements listed in the Guide (21) in their NCCPs, whereas 1 country included the fewest elements and 1 did not report any.

## 4 DISCUSSION

Based on the survey on NCCPs in EU MSs and selected countries conducted within OriON JA, most participating countries had an operational NCCP and/or a strategy, plan, or policy (cancer control documents) in place in 2024. This high level of adoption suggests a strong and sustained commitment to cancer control across Europe (24). The preparation of single, comprehensive documents in most participating countries reflects a preference for centralising strategies and initiatives within a unified framework, thereby ensuring greater consistency and coherence in policy implementation (24). Such an approach may also facilitate monitoring, revision, and implementation of the NCCPs and related cancer documents.

In contrast, nearly one-third of the participating countries addressed cancer control through several separate documents. These divergent approaches reflect differences in policy design and governance, shaped by the specific health needs, institutional arrangements, and administrative structures of individual countries. Analysis of the types of documents prepared—programmes, strategies, plans, policies—reveals substantial variation, highlighting diverse policy approaches across Europe. Nevertheless, approximately half of the participating countries prepared a programme aligned with the recommendations of the Guide (21), while many combined different types of documents (for example, programme and plan; programme, plan and strategy; programme, plan, strategy, and policy).

Regarding the duration of validity of NCCPs, most countries favour medium- to long-term planning horizons, reflecting the complexity and long-term nature of cancer control. The largest group of countries reported preparing NCCPs and/or relevant cancer control documents with an expiry date in 2030. These countries represent a forward-looking group with long-term planning horizons, potentially aiming to ensure stability and continuity in cancer control efforts. Importantly, most countries intend to develop a new NCCP or cancer control document upon expiry of their existing one. Overall, the data suggest a strong commitment among participating countries to maintaining and updating their cancer control frameworks to ensure their continued relevance and responsiveness to emerging challenges. This commitment is particularly important given that the number of cancer diagnoses and deaths is projected to increase substantially between 2024 and 2050, by 60.7% (41.9–80.6) and 74.5% (50.1–104.2), respectively (25). However, some countries reported obstacles to developing their NCCP. For example, 1 country stated that updates are needed to align its NCCP with current priorities but cited the lack of a National Cancer Mission Hub as a structural challenge. Another country stated that an NCCP exists, but there are limited opportunities for its further development or improvement.

Financing emerges as a critical challenge in European cancer control efforts. Securing consistent and sustainable funding is essential not only for implementing NCCPs and/or relevant cancer control documents but also for their long-term effectiveness. While some participating countries have made progress in securing funding for implementation, in others, financing remains a significant obstacle. Addressing funding gaps should therefore be a priority, as inadequate financing directly limits countries’ capacity to implement effective and sustainable cancer prevention, diagnosis, treatment, and care programmes. Ensuring financial security for the implementation of NCCPs may help determine the success of these important health documents and contribute to the fight against cancer. Ensuring financial security is also crucial for promoting equity in cancer care. Furthermore, as Ilbawi emphasises, comprehensive policies must support patients, caregivers, and the bereaved (26).

Data on the use of indicators for evaluating NCCPs and/or relevant cancer control documents indicate a strong overall trend towards their use, while also highlighting areas requiring further development or clarification. A multi-level evaluation approach that addresses structure, process, and outcome indicators is consistent with the recommendations of the Guide (21). Evaluation of NCCPs provides essential insights into how countries assess and refine their cancer control measures, yet this area requires greater attention. Some countries still do not evaluate their NCCPs and/or relevant cancer control documents at all, and comprehensive evaluations, including structure, process, and outcome indicators, are relatively limited. Only slightly more than one-third of participating countries reported focusing on all 3 evaluation dimensions, which provide comprehensiveness, while other countries concentrated on 1 or 2 dimensions only.

However, more than half of the participating countries included indicators in their NCCPs and/or relevant cancer control documents, reflecting a systematic approach to evaluating cancer control activities. These countries have prioritised structured evaluation mechanisms to track progress and outcomes, thereby emphasising the importance of measurable benchmarks in assessing policy implementation. Some countries described alternative evaluation approaches. One country has an established supervisory board that includes decision-makers, policymakers, and cancer patient representatives. This board provides essential oversight and strategic direction, ensuring that the plan’s objectives are met effectively and efficiently. Another country follows unique evaluation methods, including the publication of an annual implementation report.

With regard to the overall quality of NCCPs and/or other relevant cancer control documents, only 6 countries included all key elements listed in the Guide (21), whereas 1 country included the fewest elements, and 1 did not report any.

Prevention, long recognised as a key component of cancer control (27,28,29,30), is included in the NCCPs of most countries. However, important areas such as survivorship, rehabilitation, and patient empowerment, as well as financing, monitoring of cancer burden, cancer resources, and governance, are not adequately addressed. The lower coverage of these areas indicates that there remains substantial scope to strengthen the comprehensiveness and quality of NCCPs and related cancer control documents, and that there is further room for improvement in the design and comprehensiveness of cancer control documents across Europe.

In parallel with OriON JA, the OBS-PACE initiative, dedicated to advancing cancer research, care, and policy across Europe, was conducted. Case studies from across EU countries on innovative practices and policies tackling cancer at the local, national, and international levels were collected and will serve as a resource for policymakers in the field of cancer (31).

Based on the individualised shortcomings in the field of NCCPs in Europe that our analysis identified, policy recommendations for policymakers covering the area of cancer control will be prepared. They will highlight the vital importance of preparing, adopting, implementing, and evaluating high-quality NCCPs or relevant cancer control documents and support efforts to improve their quality.

## 5 CONCLUSIONS

The analysis of NCCPs prepared based on the results of the survey carried out in 34 EU MSs and selected countries within OriON JA in 2024 suggests that most European countries are actively addressing the challenge of cancer management by preparing 1 or more cancer control documents, mainly programmes or strategies. There is a noticeable preference for preparing documents with longer validity and for renewing or preparing a new NCCP before the expiry of the currently valid document, thereby ensuring continuity. Countries are aware of the importance of evaluating their NCCPs and/or relevant cancer control documents, as the majority evaluate NCCPs with the involvement of various institutions and using indicators that are also included in the NCCPs in many countries. However, funding for implementing the NCCPs remains a challenge, as it is not secured in the majority of countries. Greater attention should be devoted to the quality of NCCPs, with a stronger focus areas that are not adequately addressed, particularly survivorship, rehabilitation, patient empowerment, and complex domains such as financing, monitoring of cancer burden, cancer resources, and governance, following the Guide. High-quality, comprehensive, and well-coordinated NCCPs are important for enhancing cancer management and improving cancer outcomes and equity across Europe.
